# Signal transmission in mature mammalian vestibular hair cells

**DOI:** 10.3389/fncel.2022.806913

**Published:** 2022-07-22

**Authors:** Paolo Spaiardi, Walter Marcotti, Sergio Masetto, Stuart L. Johnson

**Affiliations:** ^1^Department of Brain and Behavioural Sciences, University of Pavia, Pavia, Italy; ^2^Department of Biology and Biotechnology, University of Pavia, Pavia, Italy; ^3^School of Biosciences, University of Sheffield, Sheffield, United Kingdom; ^4^Sheffield Neuroscience Institute, University of Sheffield, Sheffield, United Kingdom

**Keywords:** vestibular hair cells, exocytosis, ribbon synapse, non-quantal transmission, vesicle pools

## Abstract

The maintenance of balance and gaze relies on the faithful and rapid signaling of head movements to the brain. In mammals, vestibular organs contain two types of sensory hair cells, type-I and type-II, which convert the head motion-induced movement of their hair bundles into a graded receptor potential that drives action potential activity in their afferent fibers. While signal transmission in both hair cell types involves Ca^2+^-dependent quantal release of glutamate at ribbon synapses, type-I cells appear to also exhibit a non-quantal mechanism that is believed to increase transmission speed. However, the reliance of mature type-I hair cells on non-quantal transmission remains unknown. Here we investigated synaptic transmission in mammalian utricular hair cells using patch-clamp recording of Ca^2+^ currents and changes in membrane capacitance (Δ*C*_m_). We found that mature type-II hair cells showed robust exocytosis with a high-order dependence on Ca^2+^ entry. By contrast, exocytosis was approximately 10 times smaller in type-I hair cells. Synaptic vesicle exocytosis was largely absent in mature vestibular hair cells of *Ca_V_1.3* (*Ca_V_1.3^−/−^*) and *otoferlin* (*Otof^−/−^*) knockout mice. Even though Ca^2+^-dependent exocytosis was small in type-I hair cells of wild-type mice, or absent in *Ca_V_1.3^−/−^* and *Otof^−/−^*mice, these cells were able to drive action potential activity in the postsynaptic calyces. This supports a functional role for non-quantal synaptic transmission in type-I cells. The large vesicle pools in type-II cells would facilitate sustained transmission of tonic or low-frequency signals. In type-I cells, the restricted vesicle pool size, together with a rapid non-quantal mechanism, could allow them to sustain high-frequency phasic signal transmission at their specialized large calyceal synapses.

## Introduction

Vestibular hair cells detect and signal head position and movements to primary sensory neurons. The information is used by the central nervous system for driving the reflexes that control visual gaze and maintain balance and body posture. While mammals, birds, and reptiles have both type-I and type-II vestibular hair cells, fish and amphibians only have type-II (Lysakowski, 1996; Eatock et al., 1998). Type-I and type-II hair cells differ in their shape, innervation pattern, and electrophysiological properties (Eatock and Songer, 2011; Burns and Stone, 2017). Type-II hair cells are cylindrically shaped, and are contacted by several small (bouton) afferent endings (Lysakowski and Goldberg, 1997) similar to auditory inner hair cells (IHCs; Moser et al., 2006). Conversely, type-I hair cells have a distinguishing flask shape and their basolateral membrane is almost completely enveloped by a giant calyx-like expansion formed by the afferent nerve terminal (Wersäll, 1956; Goldberg, 2000; Eatock and Songer, 2011). It has been speculated that type-I hair cells and their encapsulating calyces might have evolved later on in amniotes to allow more rapid transmission of responses (Eatock, 2018), possibly required for the transition to a land-based life and the acquisition of a head moving independently of the trunk.

Signal transmission in vestibular type-I and type-II hair cells is believed to rely on the Ca^2+^-dependent quantal release of glutamate from ribbon synapses onto AMPA receptors on the bouton or calyceal afferent terminals (Dememes et al., 1995; Matsubara et al., 1999; Bonsacquet et al., 2006; Rennie and Streeter, 2006; Dulon et al., 2009; Sadeghi et al., 2014). As such, exocytosis at ribbon synapses from inner ear hair cells occurs at presynaptic active zones where voltage-gated Ca^2+^ channels are clustered (Lysakowski and Goldberg, 2008; Meyer et al., 2009; Zampini et al., 2010). Synaptic ribbons are electron-dense presynaptic organelles that tether a readily releasable pool (RRP) of vesicles docked to the presynaptic membrane and a larger secondarily releasable pool (SRP) further from the release sites (Lenzi and von Gersdorff, 2001; Matthews and Fuchs, 2010; Moser et al., 2020). These pools of vesicles allow hair cells to provide rapid and relatively inexhaustible release of neurotransmitters in response to fast and prolonged stimulation (Matthews and Fuchs, 2010; Pangrsic et al., 2018). Previous work has shown that while all immature vestibular hair cells have a large RRP and SRP of vesicles that exhibit a linear exocytotic dependence on Ca^2+^ entry (Dulon et al., 2009), mature type-II hair cells have a high-order relation between vesicle fusion and Ca^2+^ entry (no data is available for type-I: Spaiardi et al., 2020b). This change in Ca^2+^ dependence in type-II hair cells could represent a developmental adaptation of their synaptic machinery that specializes these cells for signaling low-frequency, oscillatory responses, similar to very low frequency auditory IHCs that have a comparable high-order Ca^2+^ dependence (Johnson et al., 2017). Recent studies have provided evidence that type-I hair cells are likely to use a rapid non-quantal mode, Ca^2+^ independent, signal transmission with their surrounding calyx (Yamashita and Ohmori, 1990; Holt et al., 2007; Songer and Eatock, 2013; Contini et al., 2017). However, the properties of synaptic vesicle exocytosis in mature vestibular type-I hair cells, whether they show a similar developmental change, and whether these cells can drive activity in the calyces in the absence of synaptic transmission, has yet to be determined.

In the present study, we have used patch-clamp capacitance measurements to characterize the Ca^2+^ dependence and kinetics of neurotransmitter exocytosis in mature mouse vestibular hair cells. We found that exocytosis in both type-I and type-II hair cells from the utricle displayed a high-order dependence on Ca^2+^ influx, consistent with a previous study (type-II cells only: Spaiardi et al., 2020b). However, we found that type-I hair cells, in contrast to type-II cells, showed a 10-fold smaller RRP and negligible SRP over the range of stimulus durations used. Exocytosis in both type-I and type-II hair cells was absent or largely reduced in mice lacking key components of the exocytotic machinery such as otoferlin or Ca^2+^ channels. Despite their very small Ca^2+^ -induced exocytosis, type-I hair cells were able to drive action potential activity in the calyx terminals, which was not abolished in the absence of otoferlin or Ca^2+^ channels. This supports a functional role for non-quantal transmission in type-I hair cells in sustaining prolonged signal transmission to the calyx and suggests that these cells have evolved alternative transmission mechanisms to fulfil different functional roles in the vestibular system.

## Materials and Methods

### Animals and tissue preparation

Vestibular hair cells and calyces were studied in acutely dissected C57B/6N mouse utricles from postnatal day 18 (P18) to P28, where the day of birth is P0. This is an age when the maturation of the sensory vestibular epithelium is considered complete (Burns et al., 2012; Burns and Stone, 2017). For some experiments, utricles were obtained from the following knockout mice: Ca_V_1.3 Ca^2+^ channels (*Ca_V_1.3*^−/−^: Platzer et al., 2000); Ca^2+^ sensor for vesicle fusion otoferlin (*Otof^−/−^*: Roux et al., 2006). Mice overexpressing channel rhodopsin 2 (ChR2/EYFP: The Jackson Laboratories, #024109) specifically in the sensory hair cells using *Otoferlin-Cre* mice (Kazmierczak et al., 2017; Bardhan et al., 2019) were also used. ChR2 was activated using a blue LED. Animals of either sex were killed by cervical dislocation in accordance with UK Home Office regulations under the Animals (Scientific Procedures Act) 1986 and following approval by the University of Sheffield Ethical Review Committee (180626_Mar).

Mouse utricles were dissected in the following extracellular solution (in mM): 135 NaCl, 5.8 KCl, 1.3 CaCl_2_, 0.9 MgCl_2_, 0.7 NaH_2_PO_4_, 5.6 D-glucose, 10 Hepes-NaOH. Sodium pyruvate (2 mM), MEM amino acids solution (50×, without L-Glutamine) and MEM vitamins solution (100×) were added from concentrates (Fisher Scientific, UK). The pH was adjusted to 7.5 (osmolality ~308 mmol/kg). The dissected utricles were transferred to a microscope chamber, immobilized using a nylon mesh fixed to a stainless-steel ring (Carlton et al., 2021; Jeng et al., 2021) and continuously perfused with the above extracellular solution. The utricles were observed with an upright microscope (Nikon FN1, Japan) equipped with Nomarski differential interference contrast optics (X60 water immersion objective and X15 eyepieces).

### Whole-cell electrophysiology

All whole-cell patch-clamp recordings were performed at near body temperature (34–37°C) using an Optopatchamplifier (Cairn Research Ltd, UK). Patch pipettes (3–4 MΩ) were pulled from soda glass capillaries (Hilgenberg, Germany) and coated with surf wax (Mr. ZogsSexWax, USA) to minimize the fast capacitance transient of the patch pipette. Both hair cell types from the striola or extrastriolar regions of the mouse utricle were used in this study. Access to the hair cells was gained by using a 4 μm borosilicate glass pipette filled with normal extracellular solution and connected to a syringe to apply light suction and pressure to clean the cell membrane prior to patching. For type-I hair cells, this allowed the removal of at least a small portion of the calyx. Potassium currents were recorded using a KCl-based intracellular solution containing (in mM): 131 KCl, 3 MgCl_2_, 10 Na_2_-Phosphocreatine, 5 Na_2_ATP, 5 HEPES-KOH, 1 EGTA-KOH. The pH was adjusted to 7.2 with KOH (osmolality ~295 mmol/kg).

For capacitance measurements (described below), the K^+^ currents were blocked using a CsGlutamate-based intracellular solution containing (in mM): 110 Csglutamate, 20 CsCl, 3 MgCl_2_, 1 EGTA-CsOH, 5 Na_2_ATP, 0.3 Na_2_GTP, 5 HEPES-CsOH, 10 Na_2_-phosphocreatine (pH 7.3 with CsOH; ~295 mmol/kg). For these experiments, after establishing the identity of the hair cells (type-I or type-II), the remaining K^+^currents *I_K,L_* and *I*_h_, which are not blocked by intracellular Cs^+^, were blocked by locally perfusing the hair cells with an extracellular solution containing TEA and 4-AP (in mM): 110 NaCl, 5.8 CsCl, 1.3 CaCl_2_, 0.9 MgCl_2_, 0.7 NaH_2_PO_4_, 5.6 D-glucose, 10 HEPES, 30 mM TEA, and 15 mM 4-AP (pH adjusted to 7.5 with NaOH, osmolality ~312 mmol/kg).

Voltage protocol application and data acquisition were controlled by pClamp software using a Digidata 1440A board (Molecular Devices, USA). Voltage-clamp recordings were low-pass filtered at 2.5 kHz (8-pole Bessel) and sampled at 5 kHz or 50 kHz. Data analysis was performed using Clampfit (Molecular Devices, USA) and Origin software (OriginLab, USA). Membrane potentials were corrected for the voltage drop across the series resistance (*R*_s_) and a liquid junction potential of –4 mV or –11 mV when using the KCl-based or CsGlutamate-based pipette solutions, respectively, measured between electrode and bath solutions. The average size of hair cells, as indicated by the whole-cell membrane capacitance (*C*_m_), was 4.1 ± 0.2 pF (*n* = 25) and 4.1 ± 0.2 pF (*n* = 25) for type-I and type-II cells, respectively. The isolated Ca^2+^ current recordings were corrected off-line for the linear leak current (*I*_leak_) typically calculated between –81 mV and –71 mV.

### Membrane capacitance measurements

Real-time measurement of cell membrane capacitance was performed with the “track-in” circuitry of the Optopatch amplifier (Johnson et al., 2002, 2005) using a 4 kHz sine wave voltage command (13 mV RMS amplitude) applied at the holding potential of −81 mV. The exocytosis of synaptic vesicles was measured as the change in membrane capacitance (Δ*C*_m_) produced by Ca^2+^ influx elicited by depolarizing voltage steps of variable size and duration. The sine wave used to measure real-time *C*_m_ was interrupted for the duration of the voltage steps. The capacitance signal from the Optopatch was amplified (50×), filtered at 250 Hz and sampled at 5 kHz or 50 kHz. The Δ*C*_m_ as a function of cell membrane voltage was obtained as the difference between the mean baseline capacitance signal and that measured over a 200 ms, or greater, period after each depolarizing voltage step. The Ca^2+^ dependence of vesicle exocytosis was assessed by fitting the variation in Δ*C*_m_ as a function of the peak *I*_Ca_ using the synaptic transfer function: Δ*C*_m_= c*I_Ca_*^N^*, where *c* is a scaling coefficient, and the power is *N*. The averaged *N* values reported are from fits to all individual cells tested. The fusion of vesicles from kinetically distinct vesicle pools was obtained by measuring Δ*C*_m_ in response to depolarizing voltage steps to around −11 mV, from the holding potential of −81 mV, of varying duration (2 ms to 1 s). Stimulus duration of up to 100 ms generally allow the isolation of the readily releasable pool (RRP) of vesicles when experiments are performed at body temperature and using 1.3 mM extracellular Ca^2+^ (Johnson et al., 2005, 2010). The size and release kinetics of the isolated RRP was approximated by fitting the data points from each individual cell using a single exponential function. The number of vesicles was estimated using a conversion factor of 37 aF/vesicle (Lenzi et al., 1999). The RRP and SRP measurements are not expected to be affected by endocytosis since in vestibular hair cells it is a slow process with an average time constant greater than 8 s (Dulon et al., 2009).

### Cell-attached recording

Action potential activity in calyceal terminals around type-I hair cells was recorded using the cell-attached loose patch configuration at body temperature (34°C to 37°C). Patch pipettes were made from borosilicate glass (Hilgenberg, Germany) to resistances ranging between 3 and 7 MΩ and filled with a solution similar to normal extracellular solution containing (mM): 140 NaCl, 5.8 KCl, 1.3 CaCl_2_, 0.9 MgCl_2_, 0.7 NaH_2_PO_4_, 5.6 D-glucose, 10 Hepes-NaOH (pH 7.5, ~290 mmol/kg). Seal resistances ranged from 15 to 50 MΩ. Extracellularly recorded action potentials manifest as biphasic capacitive currents resulting from the charging and discharging of the membrane below the recording pipette (Johnson et al., 2011). For some experiments, type-I hair cells were depolarized by locally perfusing a low Ca^2+^ (40 μM: buffered with HEDTA: Corns et al., 2018) extracellular solution to mimic the *in vivo* endolymph that would normally be around the hair bundle. The low Ca^2+^ solution contained (mM): 137 NaCl, 5.8 KCl, 3.9 CaCl_2_, 5.6 D-glucose, 10 Hepes-NaOH, 4 HEDTA-NaOH (pH 7.5 with NaOH, ~310 mmol/kg). To ensure the calyx spikes were being driven by hair cell depolarization, we additionally perfused the mechanotransducer channel blocker dihydrostreptomycin (100–200 μM) together with the above low Ca^2+^ extracellular solution. The AMPA receptor blocker 2,3-dioxo-6-nitro-1,2,3,4-tetrahydrobenzo[f] quinoxaline-7-sulfonamide (NBQX; 20 μM) was perfused onto the utricles in some experiments to block glutamatergic transmission.

### Statistical analysis

Differences in the mean were compared for statistical significance either by paired or unpaired Student’s two-tailed t-test. Multiple data sets were compared using repeated measures or standard one-way or two-way ANOVA followed by the appropriate multiple comparison post-test. Mean values are quoted ± s.e.m. where *P* < 0.05 indicates statistical significance.

## Results

### Identification of vestibular hair cell type in the mature mouse utricle

**Figure 1:**
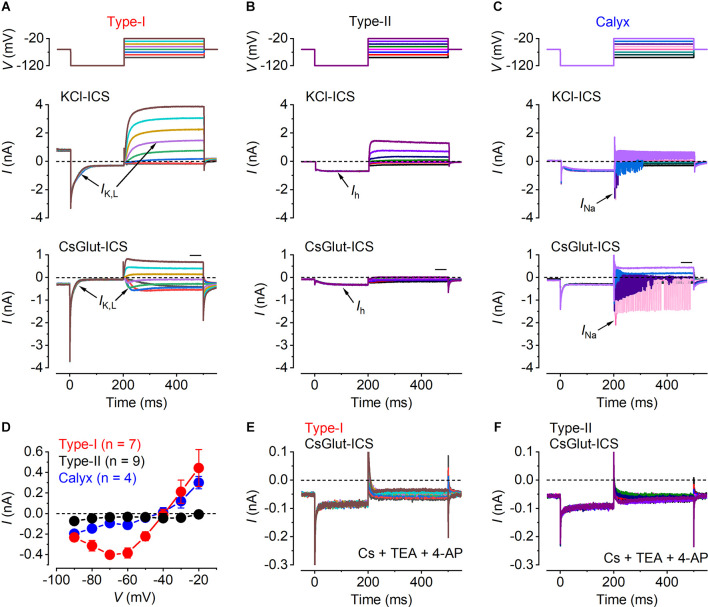
Identification of vestibular cell types. **(A–C)** Characteristic whole-cell current responses from a representative type-I hair cell **(A)**, a type-II hair cell **(B)** and a calyx afferent **(C)**. Currents were evoked by 300 ms voltage steps (upper panels), from nominally −90 mV to −20 mV, in 10 mV increments, after a hyperpolarizing 200 ms prepulse to −120 mV, from a holding potential of −60 mV. Whole-cell current responses obtained using the KCl-based (middle panels) and a CsGlutamate-based (lower panels) intracellular solution. The individual traces are color-coded to match the voltage stimulus in the upper panels. The arrows point to the characteristic currents present in the different cells, including *I*_K,L_ in type-I cells, *I*_h_ in type-II cells and *I*_Na_ in the calyx. **(D)** Mean steady-state *I-V* relation for type-I hair cells (red, *n* = 7), type-II hair cells (black, *n* = 9) and calyces (blue, *n* = 4), obtained from the CsGlut ICS recordings by plotting the steady-state current (measured in the region of the horizontal black bar above the test traces in the lower panels of **A–C**) against the corresponding membrane potential. **(E,F)** Residual currents remaining after the local extracellular perfusion of the K^+^ channel blockers CsCl (5.8 mM), TEA (30 mM), and 4-AP (15 mM; see “Methods” Section) onto a type-I and a type-II hair cell, respectively, using the CsGlutamate intracellular solution.

When characterizing the functional properties of mouse vestibular hair cells it is important not only to distinguish the type-I from the type-II hair cells but also the calyces, which are firmly attached to the type-I cells. Since hair cells were maintained in intact *ex vivo* utricles, which prevent the unambiguous identification of the two types based on their morphological differences, they were classified according to the cell’s unique complement of ion channels. Typical K^+^ current responses of a type-I, type-II and calyx recorded using a KCl-based intracellular solution are shown in Figures 1A–C (middle panels). The cells and calyx were voltage-clamped at a holding potential of −64 mV and currents were elicited by initially hyperpolarizing them to −124 mV followed by 10 mV incremental voltage steps. All recordings were obtained at body temperature (34°C to 37°C) and 1.3 mM external Ca^2+^ concentration (unless otherwise stated).

Mature type-I hair cells express, in addition to a classical delayed-rectifier K^+^ current, a characteristic outwardly rectifying K^+^ current named *I*_K,L_, which activates at unusually negative potentials and greatly reduces the input resistance of the cell at the resting membrane potential (Correia and Lang, 1990; Rennie and Correia, 1994; Ricci et al., 1996; Rüsch and Eatock, 1996a, b). Since *I*_K,L_ is fully activated at around −60 mV, type-I hair cells displayed a large outward current at the holding potential, which produced a large instantaneous inward current that fully deactivated upon hyperpolarization to −124 mV (Figure 1A, middle panel). Following its complete deactivation, *I*_K,L_ activated for potentials above −84 mV showing a delayed rectifier outward K^+^ current. Type-II hair cells showed a large hyperpolarization activated inwardly rectifying *I*_h_ current that can be identified by its delayed activation upon hyperpolarization to −124 mV (Figure 1B, middle panel). The calyx was identified by the presence of a sodium current (*I*_Na_) and transient and repetitive currents superimposed on the outward K^+^ current recordings that result from the calyx firing action potentials, triggered by depolarization above the threshold, that cannot be clamped by the amplifier (Figure 1, middle panel).

**Figure 2:**
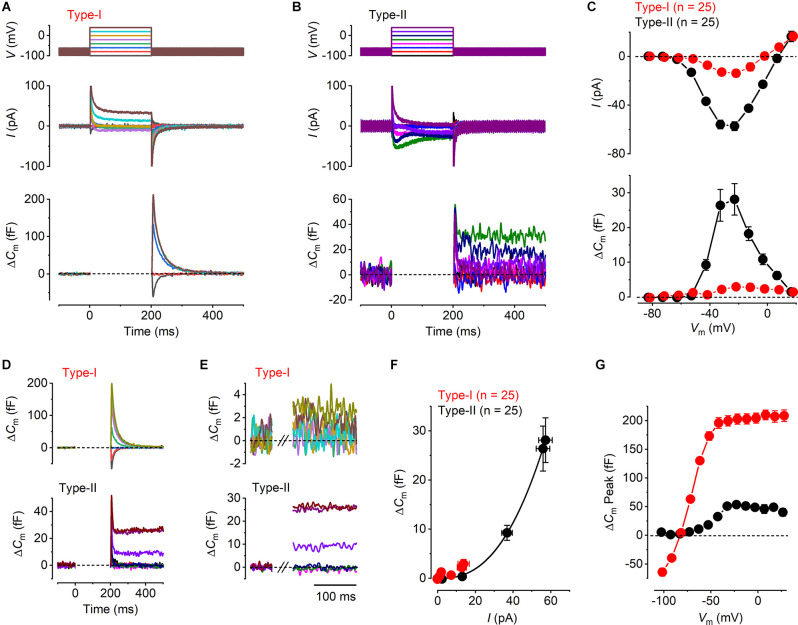
Ca^2+^ currents and Ca^2+^ dependent exocytosis in mature mouse utricular hair cells. **(A,B)** Representative *I*_Ca_ (middle panels) and the corresponding Δ*C*_m_ (lower panels) from a type-I and a type-II hair cell, respectively. The voltage protocol (upper panels) consists of a sine wave (thick solid line) interrupted for the duration of the voltage steps. *I*_Ca_ was obtained in response to 200 ms voltage steps, from −101 mV to −21 mV in 10 mV increments, from the holding potential of −81 mV. Voltage steps eliciting *I*_Ca_ produced a sustained Δ*C*_m_ in the type-II hair cells, but a large transient Δ*C*_m_ in type-I cells. The transient Δ*C*_m_ was positive or negative following depolarizing or hyperpolarizing voltage steps, respectively. **(C)** Mean peak *I-V* relation (upper panel) and the corresponding mean sustained Δ*C*_m_-*V* relation (lower panel) for type-I (red, *n* = 25) and type-II (black, *n* = 25) hair cells. **(D)** Averaged Δ*C*_m_ traces at potentials up to the peak of the *I*-*V* and Δ*C*_m_-*V* curves in **(C)** (−21 mV), for type-I (upper panel) and type-II (lower panel) hair cells. Note the different scale y-axis used for type-I and type-II hair cells. **(E)** Same average Δ*C*_m_ traces shown in **(D)** but on a magnified time scale where the transient Δ*C*_m_ has been omitted and the Δ*C*_m_ response is sustained and stable. This was the region used to measure the sustained Δ*C*_m_ response. **(F)** Synaptic transfer relations for mature type-I and type-II hair cells, which were obtained by plotting the mean Δ*C*_m_ values against the corresponding mean *I*_Ca_, between −71 mV and −21 mV from the *I*-*V* and Δ*C*_m_-*V* curves in **(B)**. Data from type-II hair cells were fitted according to the power function (see “Methods” Section). **(G)** Mean transient Δ*C*_m_ peak amplitudes in type-I (*n* = 25) and in type-II (*n* = 25) hair cells. Note that the peak transient Δ*C*_m_ was negative in type-I following voltage steps more hyperpolarized than −81 mV.

To study the Ca^2+^ dependence of synaptic vesicle exocytosis it is necessary to block the K^+^ currents to reveal the underlying *I*_Ca_. Since the K^+^ current profile is required for the prior identification of the hair cell types, we began all recordings using intracellular Cs^+^, which blocks most of the K^+^ currents but not *I*_K,L_and *I*_h_ (Rennie and Correia, 2000). Upon the identification of the different hair cell types in the utricle (Figures 1A–C, middle panel; Figure 1D), the patched cells were superfused with an extracellular solution containing TEA and 4-AP (see “Methods” Section) to block the remaining Cs^+^and *h*-type currents (Figures 1E,F). From these recordings, the isolated *I*_Ca_ was obtained by subtracting the linear leak current and the holding current (Figures 2A–C).

### Calcium dependent exocytosis in hair cells from mature mouse utricles

The exocytosis of synaptic vesicles was studied in both type-I and type-II utricular hair cells by monitoring real-time changes in membrane capacitance (Δ*C*_m_) during whole-cell patch-clamp recordings. The isolated *I*_Ca_ was recorded simultaneously to investigate the Ca^2+^ dependence of hair cells vesicle exocytosis. Representative *I*_Ca_ and Δ*C*_m_ recordings from a type-I and a type-II hair cell are shown in Figures 2A,B. The responses were evoked by depolarizing voltage steps from −101 mV in 10 mV nominal increments from the holding potential of −81 mV. Mature type-II cells showed a sustained Δ*C*_m_ following depolarizing voltage steps that were consistent with the exocytosis of synaptic vesicles, the size of which was proportional to the magnitude of the peak elicited *I*_Ca_ (Figure 2B). By contrast, all mature type-I cells (*n* = 25) showed a small sustained Δ*C*_m_ following a large transient Δ*C*_m_ that was always present immediately after the voltage steps (Figure 2A). The transient Δ*C*_m_ was either negative or positive depending on the direction of the voltage step from the holding potential, which is inconsistent with the dependence of vesicle fusion upon *I*_Ca_. Moreover, it did not decrease in response to the largest depolarizing steps that elicit little or no *I*_Ca_. The average *I*_Ca_-voltage (*I*-*V*) and Δ*C*_m_-voltage (Δ*C*_m_-*V*) relations (Figure 2C) for type-I and type-II hair cells were obtained by plotting the peak amplitude of *I*_Ca_, or the average sustained Δ*C*_m_, against the relative membrane potential of the voltage step (*V*_m_). *I*_Ca_ in both type-I and type-II hair cells showed a similar overall bell-shaped activation, which was evident from around −61 mV, reaching a maximal inward current at −21 mV. The maximal amplitude of *I*_Ca_ was smaller in type-I (−13.7 ± 3.0 pA, *n* = 25) compared to that in type-II hair cells (−57.2 ± 3.6 pA, *n* = 25) with the values being significantly different over a physiological range of potentials from −51 mV to 9 mV where there was a substantial Ca^2+^ current (*P* < 0.0001, two-way ANOVA). The similar shape of the *I*-*V* curves in both hair cell types is consistent with the current being carried predominantly by Ca_V_1.3 voltage-gated Ca^2+^ channels (Almanza et al., 2003; Bao et al., 2003; Dou et al., 2004; Masetto et al., 2005; Dulon et al., 2009; Manca et al., 2021). As for *I*_Ca_, a maximal sustained Δ*C*_m_ increase occurred at −21 mV in both type-I (3.0 ± 0.9 fF, *n* = 25) and type-II hair cells (28.1 ± 4.5 fF, *n* = 25). However, the Δ*C*_m_-*V* amplitudes were significantly larger in type-II compared to type-I hair cells over the same physiological range of potentials used for the Ca^2+^ current (−51 mV to 9 mV: *P* < 0.0001, two-way ANOVA). Even though Δ*C*_m_ responses were small for the type-I VHCs, their size was voltage-dependent being significantly different over a physiological range of voltages from −81 mV, where there should be no Ca^2+^ current or exocytosis, to −21 mV, which is the peak of the Ca^2+^ current (*P* = 0.0053; one-way ANOVA; Tukey’s post-test: value at −81 mV compared to the value at −21 mV was significant at *P* = 0.0104).

We investigated the Ca^2+^ dependence of the synaptic machinery in both hair cell types using the synaptic transfer function that describes the relation between Ca^2+^ entry and Δ*C*_m_ (Augustine et al., 1985). In order to obtain a clearer representation of the magnitude of Δ*C*_m_ at different membrane potentials, we averaged the Δ*C*_m_ traces from all cells for voltage steps up to the maximal response at −21 mV. Both the average Δ*C*_m_ responses for the type-I (Figure 2D, upper panel) and type-II (Figure 2D, lower panel) hair cells show an initial transient Δ*C*_m_ following the voltage step, which is much larger and slower in the former. This transient was followed by a sustained Δ*C*_m_, which is much larger in the type-II hair cells. The sustained Δ*C*_m_ after the transient is shown on expanded x and y axes in Figure 2E for membrane potentials between −81 mV and −21 mV. The synaptic transfer relations for type-I and type-II hair cells (Figure 2F) were obtained by plotting the average Δ*C*_m_ values against the corresponding *I*_Ca_ between the above membrane potential range. The peak *I*_Ca_ was used instead of the charge integral to minimize any possible error caused by any unblocked outward current (see Johnson et al., 2010, 2017). The synaptic transfer relation from type-II hair cells was fitted using a power value (*N*: see “Methods” Section) obtained from the fittings of 2.54 ± 0.01 (*n* = 25). Although the very small Δ*C*_m_ responses of type-I cells lead to unreliable fitting of the synaptic transfer function, we plotted the average values for a comparison with those in type-II cells. These results indicate a high-order relation between Ca^2+^ influx and neurotransmitter exocytosis in type-II cells, consistent with cooperative Ca^2+^ binding to the sensor (Dodge and Rahamimoff, 1967; Augustine et al., 1985; Zucker, 1993).

As mentioned above, both hair cell types exhibited, in addition to the sustained Δ*C*_m_ that represents Ca^2+^ dependent exocytosis, a transient component with a maximum size that was much larger in type-I compared to type-II cells, compared over all membrane potentials used since this component was evident over the entire range (*P* < 0.0001; two-way ANOVA, Figure 2G). In type-I hair cells, the transient Δ*C*_m_ peaked negatively for voltages below −81 mV and then rapidly increased for depolarizing potentials, reaching a plateau of around 200 fF for voltages greater than −51 mV (Figure 2G). By contrast, the mean transient Δ*C*_m_ response in type-II cells was much smaller, reaching a maximum of around 50 fF at −21 mV, which corresponds to the peak *I*_Ca_, followed by a decline for more depolarized potentials. Different from type-I hair cells, the transient Δ*C*_m_ in type-II cells did not show negative responses to the hyperpolarized potentials (Figure 2G).

**Figure 3:**
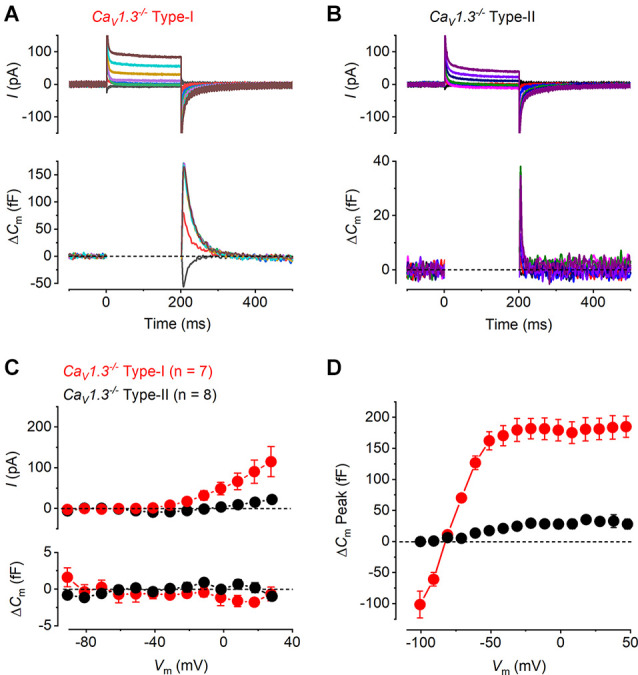
Ca^2+^ currents and Ca^2+^ dependent exocytosis in hair cells of mature Ca_V_1.3^−/−^ mouse utricles. **(A,B)** Representative *I*_Ca_ (upper panels) and the corresponding Δ*C*_m_ (lower panels) from a type-I and a type-II hair cells from *Ca_V_1.3^−/−^* mice, respectively, in response to the same voltage protocols used in Figures 2A,B. *I*_Ca_ and sustained Δ*C*_m_ responses were completely abolished in both hair cell types from *Ca_V_1.3^−/−^* mice, while the transient Δ*C*_m_ responses were still present. **(C)** Mean peak *I-V* relations (upper panel) and the corresponding mean sustained Δ*C*_m_-*V* relation (lower panel) for type-I (red, *n* = 7) and type-II (black, *n* = 8) hair cells from *Ca_V_1.3^−/−^* mice. **(D)** Mean transient Δ*C*_m_ peak amplitude in type-I (*n* = 7) and type-II (*n* = 8) hair cells from *Ca_V_1.3^−/−^* mice.

Having established the presence of differing degrees of Ca^2+^ dependent exocytosis in hair cells of the mature mouse utricle, we sought to understand whether the transient Δ*C*_m_, like the sustained component of exocytosis, was linked to Ca^2+^ entry through Ca_v_1.3 Ca^2+^ channels using *Ca_v_1.3^−/−^* mice. Representative examples of whole-cell current responses and the corresponding Δ*C*_m_ responses recorded from a mature *Ca_v_1.3^−/−^*type-I and type-II hair cells are shown in Figures 3A,B. In type-II hair cells *I*_Ca_ was largely reduced by the lack of Ca_v_1.3 Ca^2+^ channels (84%) compared to that recorded in wild-type mice, whereas that in type-I was completely absent in the *Ca_v_1.3^−/−^* mice. Neither hair cell type showed any evident sustained Δ*C*_m_ (Figure 3C). For both type-I and type-II cells the *I*_Ca_ and Δ*C*_m_ responses in the *Ca_V_1.3^−/−^* were significantly smaller than those of the wild-type (Figure 2C) over a physiological range of potentials from −51 mV to 9 mV (*I*_Ca_ and Δ*C*_m_ in both type-I and type-II, *P* < 0.0001, two-way ANOVA). This indicates that Ca_V_1.3 Ca^2+^ channels carry the vast majority of, if not the entire, Ca^2+^ current responsible for triggering synaptic vesicle exocytosis in utricle hair cells. However, the transient Δ*C*_m_ response was still present in both cell types and showed a size- and voltage dependence (Figure 3D) comparable to that observed in the wild-type hair cells (Figure 2G). The transient component in the type-II hair cells was smaller than that seen in wild-type cells due to the absence of the sustained Ca^2+^ dependent Δ*C*_m_ in hair cells from *Ca_v_1.3^−/−^*mice. This indicates that the nature of the transient Δ*C*_m_ component is not related to Ca^2+^ induced exocytosis in these cells.

**Figure 4:**
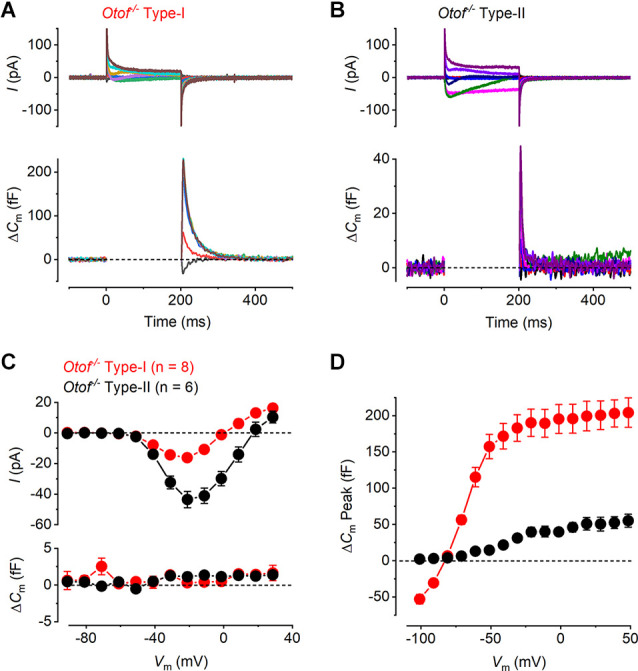
Ca^2+^ currents and Ca^2+^ dependent exocytosis in hair cells from mature otoferlin knockout mouse utricles. **(A,B)** Representative *I*_Ca_ (upper panels) and the corresponding Δ*C*_m_ (lower panels) from an Otof^−/−^ type-I and an Otof^−/−^ type-II cell, respectively, in response to the same voltage protocols used in Figures 2A,B. The *I*_Ca_ was present in both hair cell types whereas the sustained Δ*C*_m_ responses were completely abolished. The transient Δ*C*_m_ responses were still present in both type-I and type-II hair cells. **(C)** Mean peak *I-V* relation (upper panel) and the corresponding mean sustained Δ*C*_m_-*V* relation (lower panel) for type-I (−16.21 ± 1.79 pA; 0.31 ± 0.55 fF, *n* = 8) and type-II hair cells (−43.56 ± 5.34 pA, 1.13 ± 0.59 fF, *n* = 6) from *Otof^−/−^*mice. **(D)** Mean transient Δ*C*_m_ peak amplitude in type-I (*n* = 8) and type-II (*n* = 6) *Otof^−/−^* mice.

It has been previously demonstrated that otoferlin works as the main high-affinity Ca^2+^ sensor at the immature vestibular hair cells synapse, which allows efficient encoding of low-intensity stimuli (Dulon et al., 2009). To investigate the role of otoferlin at the mature vestibular hair cell synapse, we recorded whole-cell current and Δ*C*_m_ responses in both hair cell types of the utricle from mature *Otof^−/−^* mice. We found that *I*_Ca_ was present in both hair cell types from *Otof^−/−^* mice (Figures 4A,B) and, similar to that observed in the wild-type cells (Figure 2), it was smaller in type-I than in type-II hair cells. Despite the presence of a large *I*_Ca_, both hair cell types showed no sustained Δ*C*_m_ but a large transient Δ*C*_m_ (Figures 4A,B), which is similar to that observed in the *Ca_v_1.3^−/−^* cells (Figure 3). The transient Δ*C*_m_ was again much larger and slower in type-I than that in type-II hair cells. The mean *I-V* and the corresponding sustained Δ*C*_m_-*V* curves for both hair cell types from mature *Otof^−/−^* show that at the membrane potential that elicited the maximal *I*_Ca_(−21 mV), the average sustained Δ*C*_m_ was almost negligible (Figure 4C). For both type-I and type-II cells the Δ*C*_m_ responses in the *Otof^−/−^* were significantly smaller than those of the wild-type between −51 mV and 9 mV (type-I, *P* = 0.015; type-II, *P* < 0.0001, two-way ANOVA). This is consistent with otoferlin being the main exocytotic Ca^2+^ sensor in mature utricle hair cells. The transient Δ*C*_m_ responses remained present in utricular hair cells from *Otof^−/−^* mice (Figure 4D), the size and time course of which were comparable to that recorded from hair cells of *Ca_v_1.3^−/−^*mice (Figure 3).

### Dynamics of vesicle pool recruitment in mature mouse utricular hair cells

**Figure 5:**
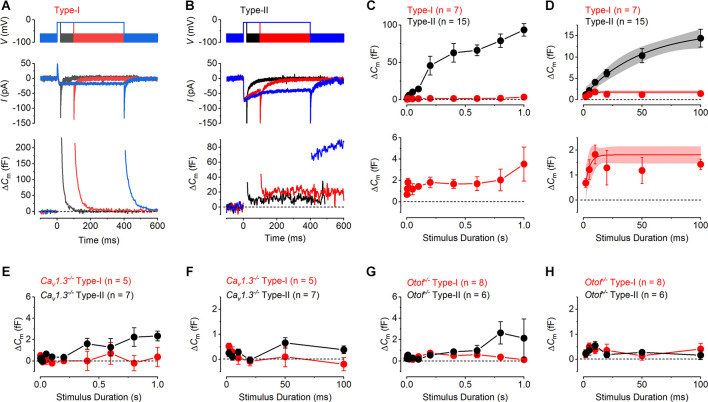
Vesicle pool dynamics in mature mouse utricular hair cells. **(A,B)** Calcium current (*I*_Ca_: middle panels) and corresponding Δ*C*_m_ (bottom panels) responses recorded from a type-I **(A)** and a type-II **(B)** hair cell, elicited by applying voltage steps to −11 mV of 20, 100, and 400 ms in duration (upper panels). **(C)** Average sustained Δ*C*_m_ responses from type-I (*n* = 6) and type-II (*n* = 15) hair cells to the different stimulus durations (upper panel). Type-II hair cells displayed a triphasic profile of vesicle pool release dynamics, representing the release of the RRP, followed by the release of a larger SRP and a final cytoplasmic pool component. By contrast, type-I hair cells showed a very small sustained Δ*C*_m_ component even at the longest stimulus durations (1 s). The lower panel shows the same values plotted in the upper panel for the type-I hair cell but on an expanded y-axis. **(D)** RRP component in both hair cell types (upper panel) could be fitted with a single exponential, which is also shown on an expanded y-axis for type-I hair cells in the lower panel. **(E)** Average sustained Δ*C*_m_ responses from type-I (*n* = 5) and type-II (*n* = 7) hair cells from *Ca_V_1.3^−/−^* mice to different stimulus durations up to 1 s. **(F)** Average Δ*C*_m_ responses as in **(E)** but showing the initial 100 ms that would represent the RRP. **(G)** Average sustained Δ*C*_m_ responses from type-I (*n* = 8) and type-II (*n* = 6) hair cells from *Otof^−/−^*mice to different stimulus durations up to 1 s. **(H)** Average Δ*C*_m_ responses as in **(G)** but showing the initial 100 ms.

The recruitment of different ribbon synaptic vesicle pools in mouse hair cells from the mature utricle was investigated by measuring *I*_Ca_ and Δ*C*_m_ in response to depolarizing voltage steps that ranged in duration from 2 ms to 1 s. The use of progressively lengthening voltage steps has been shown to recruit distinct vesicle pool populations in auditory hair cells (Moser and Beutner, 2000; Johnson et al., 2005, 2017) and type-II vestibular hair cells (Dulon et al., 2009; Spaiardi et al., 2020b). Typical *I*_Ca_ and Δ*C*_m_ responses from a type-I and a type-II hair cell of wild-type mice, which were elicited using voltage steps of 20, 100, and 400 ms in duration, are shown in Figures 5A,B, respectively. As already described above, type-I hair cells showed an initial large transient Δ*C*_m_ following the depolarizing voltage step and a very small sustained Δ*C*_m_. By contrast, type-II cells showed a substantial sustained increase in Δ*C*_m_, which increased with voltage step duration.

To investigate the progressive recruitment of discreet vesicle pools we plotted the mean sustained increase in Δ*C*_m_ (after the transient Δ*C*_m_) at −11 mV against the stimulus duration. The kinetics of vesicle pool release in type-II hair cells showed an approximately triphasic pattern of secretion (Figure 5C). The initial component, which was triggered by voltage steps up to around 100 ms, is commonly ascribed to the release of the readily-releasable pool (RRP) of the small fraction of vesicles densely packed near the active zone and available for immediate release (Moser and Beutner, 2000; Johnson et al., 2005, 2017; Dulon et al., 2009). The RRP was followed, for longer voltage steps from 200 ms to 600 ms, by a larger sustained secondary component, corresponding to the recruitment of the secondary releasable pool (SRP) of vesicles. The SRP represents a group of vesicles docked further up the ribbon, away from the active zones and the Ca^2+^ channels that becomes recruited as the RRP becomes depleted (von Gersdorff et al., 1996; von Gersdorff and Matthews, 1999; Voets et al., 1999). The third component is likely to represent the recruitment of vesicles from a cytoplasmic pool that then begins to refill the ribbon. This component is more linear and was evident from around 600 ms to 1,000 ms. In the type-II hair cells, the mean RRP kinetic component could be well approximated with a single exponential function, revealing a maximum Δ*C*_m_ increase of 16.3 ± 3.0 fF (*n* = 15, Figure 5D), and a time constant of 48.9 ± 2.6 ms and a total number of 440 vesicles (37aF per vesicle: Lenzi et al., 1999). The calculated initial maximum release rate for the RRP in type-II hair cells was around 330 fF/s or 9,000 vesicles/s, which considering a mean of seven to nine ribbons per hair cell (Dulon et al., 2009) gives an estimation of 49–62 synaptic vesicles per ribbon (SV/ribbon). These values were slightly smaller than those previously reported in immature type-II hair cells (68–88 SV/ribbon: Dulon et al., 2009), but analogous to those reported in mature mouse cochlear inner hair cells (24–64 SV/ribbon; Johnson et al., 2005; Khimich et al., 2005; Nouvian et al., 2006). The Δ*C*_m_ of the second component of vesicle pool release, the SRP, was approximated with a single exponential with a maximum value of 66.4 ± 9.5 fF (*n* = 15) and a time constant of 109.2 ± 71.8 ms. The size of the SRP of vesicles in isolation, therefore, equates to 50.1 fF or 1,354 vesicles. This gives an estimation of between 150 and 193 SRP SV/ribbon for type-II, analogous to the values previously reported for immature and adult type-II hair cells (Dulon et al., 2009; Spaiardi et al., 2020b). The recruitment of vesicles from a cytoplasmic pool reached a value of 93.50 ± 8.39 fF for 1 s stimulation, corresponding to the release of 2,520 vesicles.

In type-I hair cells, the sustained Δ*C*_m_ responses were comparatively very small throughout the entire range of stimulus durations used (Figure 5C). Despite this, there was evidence of an initial component of release that reached a maximum of up to around 600 ms stimulation. A single exponential fit to the responses from 2 ms to 600 ms gave a maximal Δ*C*_m_ of 1.81 ± 0.17 fF (*n* = 7), which equates to 49 vesicles, and a time constant of 3.7 ± 3.4 ms. This gave an initial maximum vesicle release rate of around 490 fF/s or 13,220 vesicles/s in type-I hair cells, which is comparable to that of type-II cells. Assuming there are seven equally functioning ribbon synapses in each type-I cell (Dulon et al., 2009; Vincent et al., 2014) then this represents the release of seven vesicles/ribbon. The more linear increase for the longest stimuli above 600 ms with the maximal Δ*C*_m_ response of 3.95 ± 1.88 fF occurring at 1 s stimulation, corresponding to the release of 107 vesicles, about 20 times less than in type-II. The vesicle pool release kinetics were also investigated in type-I and type-II hair cells from *Ca_v_1.3^−/−^* (Figures 5E,F) and *Otof^−/−^*mice (Figures 5G,H). Both the RRP and the SRP were almost completely abolished in both hair cell types, and only small Δ*C*_m_ responses were seen for the longer stimuli in type-II cells in both *Ca_v_1.3^−/−^* and *Otof^−/−^*mice. The small Δ*C*_m_ responses of wild-type type-I hair cells were significantly different from those recorded in *Ca_v_1.3*^−/−^ and *Otof^−/−^* mice when compared from 2 ms to 1 s (*P* < 0.0001 for each, two-way ANOVA). The same was true when values from 2 ms to 100 ms were compared, which represents the RRP of vesicles for type-II cells (Figure 5D). The Δ*C*_m_ responses were not significantly different between type-I cells recorded from *Ca_v_*1.3^−/−^ and *Otof^−/−^* mice when compared over both stimulus duration ranges (*P* > 0.05 for each, two-way ANOVA).

The above findings indicate that Ca^2+^ dependent exocytosis was very small in mature type-I hair cells compared to type-II cells and that it was almost completely abolished in both cell types when either Ca_V_1.3 Ca^2+^ channels or otoferlin were absent.

### Signal transmission from type-I hair cells to their calyx nerve terminals

It is well established that mutations in key synaptic proteins, including Ca_V_1.3 Ca^2+^ channels and otoferlin, cause deafness but only minor balance defects at worst (Platzer et al., 2000; Brandt et al., 2003; Dou et al., 2004; Roux et al., 2006; Dulon et al., 2009). One hypothesis for this discrepancy is that signal transmission from the type-I vestibular hair cells is preserved in the absence of Ca^2+^-dependent exocytosis due to the presence of non-quantal transmission between these cells and their afferent calyx terminal (Eatock, 2018).

**Figure 6:**
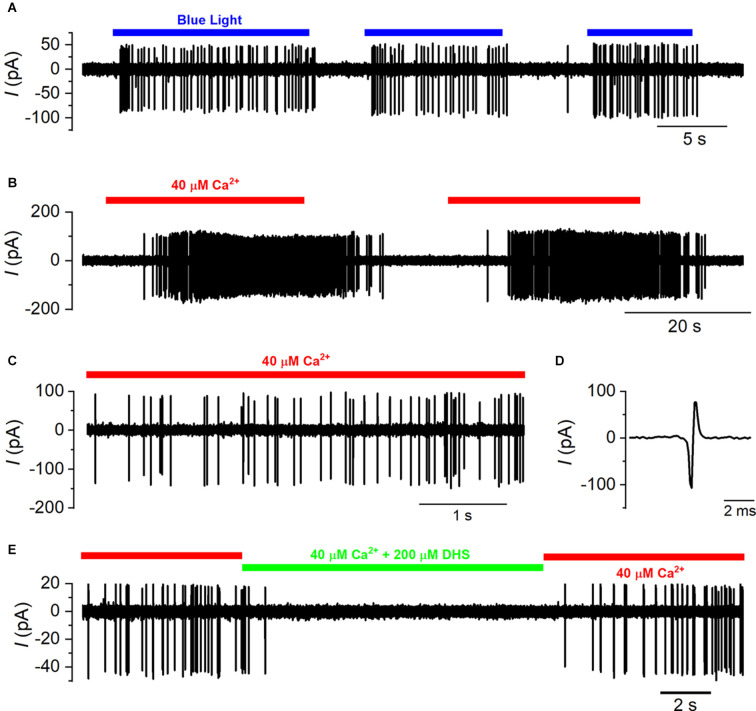
Spiking activity in the calyx is driven by type-I hair cell depolarization. **(A)** A typical example of a cell-attached recording from a calyx in the mouse utricle where spiking activity was elicited in *ChR2^fl/fl^; Otof-cre* ^+^*^/^*^−^ mice by the repeated exposure of the utricle to blue light. **(B)** Calyx spiking was elicited in wild-type mice by the local perfusion of an extracellular solution containing 40 μM Ca^2+^ (indicated by the red bar) instead of normal extracellular solution that contained 1.3 mM Ca^2+^. Spiking activity was repeatedly elicited by the low Ca^2+^ solution. **(C)** Expanded view of the spiking activity in the presence of 40 μM Ca^2+^ from panel **(B)**. **(D)** A single extracellular spike that results from an action potential within the calyx that causes a biphasic current transient that is recorded from the membrane within the recording pipette. **(E)** Calyx spiking activity triggered by 40 μM Ca^2+^ is reversibly suppressed by the additional perfusion of the MET channel blocker DHS (200 μM; indicated by the green bar).

Action potentials in the calyx terminal were recorded using the cell-attached loose patch configuration (Figure 6A). We initially performed cell-attached recordings from the calyx using mice expressing channel rhodopsin 2 specifically in the hair cells (*ChR2^fl/fl^;Otof-cre* ^+^*^/^*^−^: see “Methods” Section). When the type-I hair cells were exposed to blue light and depolarized, the calyx spiking activity increased from 2.32 ± 1.67 Hz to 37.68 ± 5.95 Hz (*n* = 6; *P* = 0.0006, paired *t*-test; Figure 6A). These experiments showed that spontaneous spiking activity in the calyx was very low in our *in vitro* recording conditions and could be reliably and repeatably elicited by depolarizing the presynaptic type-I VHC.

In mice that lacked channel rhopsin 2 expression, action potential activity in the calyx was elicited by locally perfusing an endolymphatic-like solution containing 40 μM Ca^2+^, instead of the usual 1.3 mM Ca^2+^, onto the utricle to depolarize presynaptic type-I VHCs (Figure 6B). Since Ca^2+^ acts as a permeant blocker of the mechanoelectrical transducer (MET) channel and also decreases its resting open probability (Crawford et al., 1991; Corns et al., 2014), low extracellular Ca^2+^ concentration would increase the hair cell resting mechanoelectrical transducer current and depolarize type-I cells to somewhere close to their *in vivo* resting potential. Repeated application of the low Ca^2+^ solution reliably and reversibly increased the spiking activity in the calyx terminal (Figures 6B,C). The spike rate increased from 0.08 ± 0.07 Hz in normal 1.3 mM Ca^2+^ to 20.98 ± 2.62 Hz (*n* = 19) in the presence of 40 μM Ca^2+^ in wild-type cells (*P* < 0.0001; paired *t*-test). To prove that the increase in calyx spiking was caused by the depolarization of type-I hair cells, and not by an indirect effect of the low Ca^2+^ solution on the calyx itself, we co-applied the aminoglycoside antibiotic dihydrostreptomycin (DHS) that is a well-characterized MET channel blocker in both the cochlear and vestibular hair cells (Kroese and van den Bercken, 1980, 1982; Kroese et al., 1989; Marcotti et al., 2005). Dihydrostreptomycin reversibly reduced the spiking activity in the presence of 40 μM Ca^2+^ (low Ca^2+^: 21.80 ± 4.60 Hz; low Ca^2+^ + DHS: 0.49 ± 0.34 Hz, *n* = 6, *P* = 0.007: paired t-test, Figure 6E).

**Figure 7:**
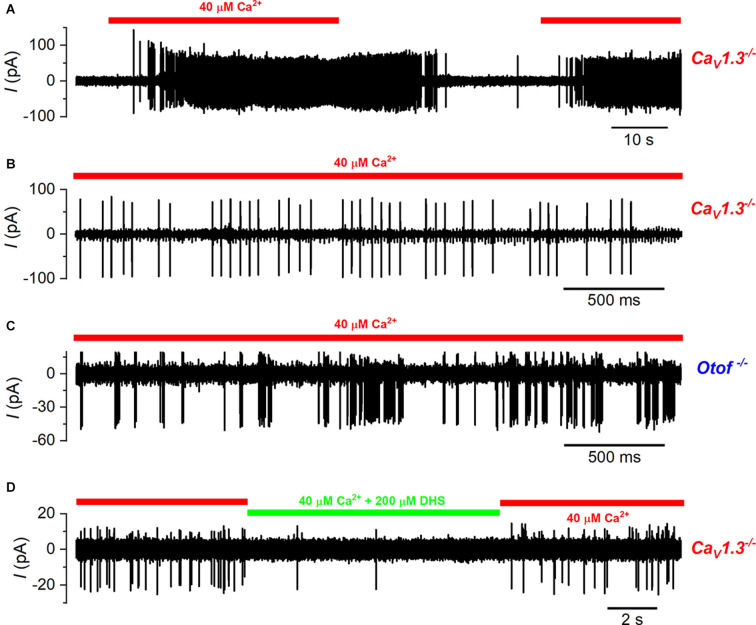
Calyx spiking activity is elicited by type-I hair cell depolarization in *Ca_V_1.3^−/−^* and otoferlin^−/−^ mice. **(A)** A typical example of a cell-attached recording from a calyx of a *Ca_V_1.3^−/−^* mouse. As for wild-type cells, spikes were elicited by the repeated local perfusion of 40 μM Ca^2+^ (indicated by the red bar) instead of normal extracellular solution that contained 1.3 mM Ca^2+^. **(B)** Expanded view of the spiking activity in the presence of 40 μM Ca^2+^ from panel **(A)**. Note that in this calyx there were large spikes and small spikes that were of a higher frequency and likely due to the branching of this fiber to contact another type-I hair cell. **(C)** Action potentials in the calyx during the perfusion of 40 μM Ca^2+^ in an *Otof^−/^*^−^ mouse. **(D)** Calyx spiking activity triggered by 40 μM Ca^2+^ in Ca_V_1.3^−/−^ mice is reversibly suppressed by the additional perfusion of the MET channel blocker DHS (200 µM; indicated by the green bar).

Having established that we could reliably measure signal transmission from the type-I hair cells to the calyx with the application of a low Ca^2+^ solution, we investigated whether it was disrupted in mice lacking the Ca^2+^ current and Ca^2+^-dependent exocytosis (*Ca_V_1.3^−/−^* and *Otof^−/−^*). As shown in the wild-type animals (Figure 6), we were able to record spiking activity from the calyx terminals that was reliably and reversibly increased in frequency with the application of the low Ca^2+^ solution in both *Ca_V_1.3^−/−^* (Figures 7A,B) and *Otof^−/−^* mice (Figure 7C). The average spike frequency in the presence of 40 μM Ca^2+^ was 15.32 ± 2.12Hz (*n* = 16) in the *Ca_V_1.3^−/−^*, and 74.89 ± 18.52 Hz (*n* = 3) in the *Otof^−/−^*mice. Similar to wild-type mice, the application of DHS also reduced the spiking activity in the calyces of *Ca_V_1.3^−/−^* mice in the presence of 40 μM Ca^2+^ (low Ca^2+^: 15.57 ± 4.41 Hz; low Ca^2+^ + DHS: 2.60 ± 1.31 Hz, *n* = 5, *P* = 0.015: paired t-test, Figure 7D).

**Figure 8:**
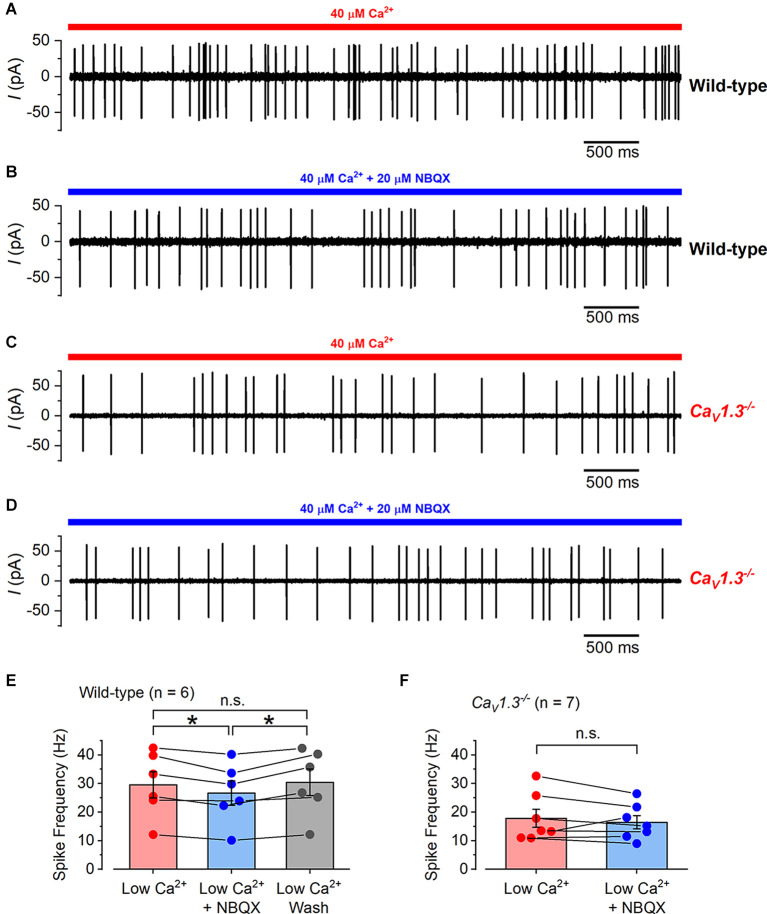
The effect of NBQX application on calyx spiking activity in wild-type and *Ca_V_1.3^−/−^* mice. **(A)** A typical example of a cell-attached recording from a calyx in a wild-type mouse utricle where spikes were elicited by the local perfusion of 40 μM Ca^2+^ (indicated by the red bar). **(B)** Cell-attached spiking activity from the same calyx in **(A)** but in the presence of 20 μM NBQX together with 40 μM Ca^2+^ (indicated by the blue bar). **(C)** Cell-attached spiking activity from a calyx of a Ca_V_1.3^−/−^ mouse in the presence of 40 μM Ca^2+^. **(D)** Spiking activity from the same calyx in **(C)** in the presence of 20 μM NBQX together with 40 μM Ca^2+^. **(E)** The average cell-attached spike frequency in wild-type calyces in low Ca^2+^ (40 μM; red), low Ca^2+^ with NBQX (20 μM; blue), and after low Ca^2+^ wash (gray). The columns show the average values with error bars and the individual data points are shown as scatter plots on top with connecting bars to the linked values from the same cell. The values in NBQX were slightly but significantly smaller than those in low Ca^2+^ and washout (*P* = 0.03 and *P* = 0.02, respectively; repeated measures one-way ANOVA Tukey post-test). There was no significant difference between the values in low Ca^2+^ compared to washout (*P* = 0.17). **(F)** The average cell-attached spike frequency in Ca_V_1.3^−/−^ calyces in low Ca^2+^ (40 μM; red), and low Ca^2+^ with NBQX (20 μM; blue). The values are shown as in **(E)** and there was no significant difference between the data.

In some experiments, we perfused the specific AMPA receptor blocker NBQX (20 μM) to prevent or reduce the glutamate-dependent signal transmission from the type-I hair cells to the calyx terminals (Dulon et al., 2009; Sadeghi et al., 2014). When NBQX was perfused together with the low Ca^2+^ solution onto the type-I hair cells of wild-type mice (Figures 8A,B), the calyx spike frequency was slightly but significantly reduced from 29.59 ± 4.60 Hz (in low Ca^2+^) to 26.67 ± 4.25 Hz (low Ca^2+^ with NBQX), which returned to 30.43 ± 4.62 Hz (low Ca^2+^ washout; *n* = 6; overall *P* = 0.007 repeated measures one-way ANOVA; Figure 8E). When we repeated these experiments on type-I hair cells from *Ca_V_1.3^−/−^*mice (Figures 8C,D), we found that NBQX did not significantly affect the calyx spiking activity, being 17.86 ± 3.14 Hz in low Ca^2+^ and 16.46 ± 2.30 Hz (*n* = 7; *P* = 0.34, paired *t*-test) in the presence of low Ca^2+^ and NBQX (Figure 8F). Spike rates were calculated from recording periods ranging from 10 s to 100 s, with the same duration used for low Ca^2+^, and low Ca^2+^ with NBQX. NBQX was applied for up to 4 min without spikes disappearing, and on a few occasions, it was applied up to four times for 2–3 min each, for up to 30 min of recording from each cell. These data show that signal transmission between type-I hair cells and their afferent calyx terminals is relatively normal even in the absence of the classical Ca^2+^-dependent exocytosis of synaptic vesicles.

## Discussion

We have investigated the synaptic function of mature mammalian vestibular type-I and type-II hair cells using physiological recording conditions (1.3 mM extracellular Ca^2+^ and body temperature) and mice lacking key components of the vesicle release machinery. We found that mature type-II hair cells showed robust sustained Ca^2+^-dependent exocytosis of synaptic vesicles with a high-order dependence on Ca^2+^ entry. By contrast, the sustained Ca^2+^-dependent exocytotic component in mature type-I hair cells was approximately 10 times smaller compared to type-II hair cells. The size of the initial RRP kinetic component of vesicle release was correspondingly 10 times smaller in type-I compared to type-II, in wild-type cells, with little evidence of an SRP component in type-I. Exocytosis of the RRP in both hair cell types was absent in *Ca_V_1.3^−/−^*and *Otof^−/−^* mice with only a very small degree of exocytosis remaining in type-II hair cells for longer stimulus durations. Type-I hair cells, despite the very small Ca^2+^-dependent exocytosis in wild-type mice, or its absence in *Ca_V_1.3^−/−^* and *Otof^−/−^* mice, were able to trigger action potential activity in their postsynaptic calyces. This supports a functional role for non-quantal transmission in these cells and could explain the apparent lack of a substantial vestibular phenotype in *Ca_V_1.3^−/−^* and *Otof^−/−^* mice (Platzer et al., 2000; Roux et al., 2006).

### Differences in exocytosis between type-I and type-II hair cells

All hair cells recorded in this study showed an initial transient Δ*C*_m_ component immediately after the voltage step, which decayed completely and was followed by a sustained Δ*C*_m_ component lasting the full duration of the recording. The transient and sustained Δ*C*_m_ components in type-II hair cells were very similar to those described in other hair cells, such as those in the auditory system (Moser and Beutner, 2000; Johnson et al., 2002). The sustained Δ*C*_m_ was dependent on Ca^2+^ and is the component that reflects the exocytosis of synaptic vesicles (Parsons et al., 1994; Moser and Beutner, 2000; Johnson et al., 2002, 2005). On the other hand, the transient Δ*C*_m_ component in type-II hair cells was relatively small, rapid and Ca^2+^-independent, and was due to the Δ*C*_m_ signal recovering from a large membrane resistance drop during the voltage step (Johnson et al., 2002). By contrast, type-I hair cells showed a very large and slow transient Δ*C*_m_ component, which was voltage-dependent until saturation, Ca^2+^-independent, and unaffected in *Ca_V_1.3^−/−^* and *Otof^−/−^* mice, indicating that it is not associated with Ca^2+^-dependent exocytosis. This large Δ*C*_m_ transient could be produced by intra-membrane charge movements, as previously suggested in adrenal chromaffin cells (Horrigan and Bookman, 1994). This transient could be due to gating charge movement of the *I*_K,L_ channels since we never observed this phenomenon in type-II hair cells, and it was elicited at hyperpolarized voltages where *I*_K,L_ is the only available current. Furthermore, the size of Δ*C*_m_ saturates at voltages where* I*_K,L_ is fully activated or deactivated (Spaiardi et al., 2017). Although this transient Δ*C*_m_ response has never been reported for type-I hair cells (Dulon et al., 2009; Vincent et al., 2014), this might be due to the younger age of mice investigated previously. Both vestibular hair cell types showed a sustained Δ*C*_m_ component that was evident immediately after the transient component.

### Calcium-dependent exocytosis in mature hair cells from the utricle

We found that *I*_Ca_ was four times larger in mature type-II than in type-I hair cells, which is consistent with that previously reported for immature utricular hair cells (Dulon et al., 2009). *I*_Ca_ triggered a sustained Δ*C*_m_ component in both hair cell types, reflecting the exocytosis of synaptic vesicles, but was around 10 times larger in type-II hair cells compared to that in type-I cells. Despite these differences, the Ca^2+^-dependence of vesicle release in type-II hair cells was high-order, consistent with our previous findings in these mature cells (Spaiardi et al., 2020b). Vestibular type-II hair cells from the immature mouse utricle (P4-P9) have been shown to elicit a linearly Ca^2+^-dependent exocytosis (Dulon et al., 2009), indicating that these cells are likely to undergo a substantial physiological change at the synapses during development, which appears to be opposite to that occurring in the auditory hair cells. In the mouse cochlea, the exocytotic Ca^2+^-dependence in IHCs that respond to acoustic frequencies above a few kHz changes from high-order to linear at the onset of hearing function at around P12 (Brandt et al., 2005; Johnson et al., 2005, 2017). However, low-frequency IHCs retain a high-order Ca^2+^ dependence even in the adult cochlea (Johnson et al., 2017). The linear relation in mature high-frequency IHCs has been shown to be dependent on the presence of the Ca^2+^ sensor synaptotagmin 4 (Syt-4), and not otoferlin, which becomes expressed in these cells at the onset of function (Johnson et al., 2010). Syt-4 seems not to be functionally involved where exocytosis shows a high-order Ca^2+^ dependence, such as in immature IHCs, low-frequency adult IHCs (Johnson et al., 2008, 2010), and mature type-II hair cells (Spaiardi et al., 2020b).

Using mice lacking molecular components of the synaptic machinery (*Ca_V_1.3^−/−^* and *Otoferlin^−/−^*) we found that Ca^2+^-dependent exocytosis was abolished or largely reduced in both type-I and type-II hair cells. The proportion of *I*_Ca_ carried by Ca_V_1.3 Ca^2+^ channels (type-II: 84%; type-I: 100%) was greater than that previously reported in immature (P1-P10) hair cells from the utricle (~50%: Dou et al., 2004), which further suggests the presence of developmental change in their synaptic machinery. We recently found that a much larger proportion of *I*_Ca_ was carried by Ca_V_1.3 channels in type-I cells from the crista ampularis of older mice (up to P20, Manca et al., 2021). A developmental change in the VHC synaptic machinery is highlighted by findings from immature *Otof*^−/−^ mice, where exocytosis was shown to be less otoferlin-dependent in VHCs than we have found here in mature cells, especially for type-II cells (Dulon et al., 2009). A developmental change in the Ca^2+^ sensor of exocytosis is similar to cochlear IHCs where there is a transition from an otoferlin-independent to an otoferlin-dependent mechanism in early postnatal mice (Beurg et al., 2010). The large number of Ca^2+^ binding C2 domains on otoferlin is likely to contribute to the high-order Ca^2+^ dependence of exocytosis in mature vestibular type II hair cells, as well as being related to the many functional roles that otoferlin serves in the synaptic vesicle cycle (PangrPangršič et al., 2012; Moser and Starr, 2016). The co-expression of otoferlin with other synaptic Ca^2+^ sensors seems to tailor the Ca^2+^-dependence of the machinery to fulfil the different functional roles of hair cells depending on specific requirements, which are dictated by their location and stage of development (Roux et al., 2006; Dulon et al., 2009; Beurg et al., 2010; Johnson et al., 2010; Vincent et al., 2014). While a linear Ca^2+^-dependence is suited for precisely encoding graded receptor potentials over a wide dynamic range in high-frequency auditory hair cells, a higher-order relation could be more beneficial for representing phasic receptor potentials with high speed and fidelity (Johnson, 2015; Johnson et al., 2017), which is a requirement of low-frequency IHCs and vestibular hair cells that drive the fastest reflex in the body, the vestibulo-ocular reflex.

### Vesicle pool size and release kinetics in mature hair cells from the utricle

Synaptic vesicle pool recruitment in type-II hair cells was consistent with Ca^2+^ influx triggering the fusion of a substantial readily releasable pool (RRP) of vesicles, followed by the recruitment of a larger secondary releasable pool (SRP), similar to that previously observed in mammalian cochlear hair cells (Moser and Beutner, 2000; Johnson et al., 2005; Beurg et al., 2008), immature vestibular type-I and II hair cells (Dulon et al., 2009; Vincent et al., 2014), and hair cells from lower vertebrates (Parsons et al., 1994; Edmonds et al., 2004; Schnee et al., 2005). By contrast, we found that the RRP in mature type-I hair cells was around an order of magnitude smaller than that of mature type-II cells, and potentially a small SRP for stimuli greater than 600 ms. Since both hair cell types appear to have a similar size of RRP and SRP in the immature utricle (Dulon et al., 2009), the smaller pool of vesicles in mature type-I hair cells is again likely to be linked to a developmental reduction in the numbers of ribbon synapses or a decline in the number of release sites recruited for a given stimulus. The latter is more likely since both hair cell types in both the utricle and cristae have similar numbers of synaptic ribbons, ranging from around seven to nine in the immature utricle (Dulon et al., 2009) to around 17–20 in mature cristae (Lysakowski and Goldberg, 1997; Sadeghi et al., 2014). Even though the vesicle pools were functionally smaller in type-I hair cells, the initial maximum release rate of the RRP was comparable between the two cell types, suggesting that the release of the RRP would be able to support rapid signaling for brief pulses of stimulation.

The different vesicle pool size between the mature vestibular hair cell types is likely to reflect the architecture of the synaptic contacts around the individual cells. Type-II hair cells are innervated by multiple afferents that form a bouton synapse with a presynaptic ribbon, a similar arrangement to cochlear IHCs (Moser et al., 2006) and as such have comparable vesicle pool sizes and release kinetics. By contrast, the basolateral membrane of type-I hair cells is completely enveloped by a single giant calyx terminal that receives the output from the hair cells’ entire complement of ribbon synapses. Therefore, the number of vesicles released in the RRP of a mature type-I onto a single calyx (about 50 vesicles) is likely to be comparable to the amount of RRP vesicles released by type-II cells onto a single afferent terminal (also about 50 vesicles, assuming nine ribbons per cell and that each releases vesicles with equal probability, i.e., 440 total RRP vesicles/nine ribbons). Postsynaptic recordings from mature type-I hair cell calyces (Dulon et al., 2009; Sadeghi et al., 2014) have shown that the rate of vesicle release is similar to mature IHCs (Grant et al., 2010) but the size of individual events was much smaller, suggesting that more vesicles are likely to be released onto single boutons than the entire calyx. The capacity of individual release events to elicit action potentials in the calyx of type-I hair cells remains to be determined. It is possible that the very restricted volume of the synaptic cleft between the type-I hair cells and the calyx, which can be as narrow as 7 nm in places (Gulley and Bagger-Sjöbäck, 1979), and the fact that glutamate can spill over between release sites (Sadeghi et al., 2014), provides a high synaptic gain that maximizes the effect of glutamate release on the postsynaptic terminal. This could also explain the apparent lack of SRP in mature type-I hair cells since excessive glutamate release could easily become excitotoxic to the calyx or overstimulate the terminal during prolonged stimulation. It is therefore possible that the large SRP in type-I hair cells could have been replaced by an alternative non-quantal mechanism (Eatock, 2018).

### Signal transmission from type-I hair cells to their afferent calyx terminals

The major aim of the present study was to establish the importance of quantal and non-quantal transmission at vestibular hair cell synapses. We found that the calyces showed an increase in action potential activity following depolarization of the type-I hair cells to somewhere close to their *in vivo* resting potential, either by the activation of ChR2 or by the perfusion of an *in vivo*-like endolymphatic Ca^2+^ concentration in wild-type as well as in *Ca_V_1.3^−/−^* and in *Otof^−/−^* mice. Moreover, the AMPA receptor blocker NBQX, which has been shown to rapidly and reversibly reduce quantal synaptic transmission in the type-I calyx (Sadeghi et al., 2014), was able to significantly reduce the calyx spiking activity elicited by low-Ca^2+^ perfusion in wild-type, but not *Ca_V_1.3^−/−^* mice. A reduction in calyx spiking activity with NBQX was also previously reported (Dulon et al., 2009), although there was an almost complete block of the activity during long-lasting applications (10–15 min—with no washout).

Overall, these findings are consistent with a significant, but non-crucial, role of Ca^2+^-dependent glutamate exocytosis in signal transmission at the calyx synapse, which might explain the apparent lack of a substantial vestibular phenotype in *Ca_V_1.3^−/−^* and *Otof^−/−^* mice (Platzer et al., 2000; Roux et al., 2006). Since we were mainly investigating type-I cell signal transmission around the likely *in vivo* resting potential, resulting from the perfusion of an endolymphatic Ca^2+^ concentration, it is possible that as the cell becomes increasingly stimulated the relative contributions of quantal and non-quantal transmission could change.

The mechanism of non-quantal transmission likely involves K^+^ exiting the basolateral membrane of the type-I hair cells directly depolarizing the calyx by changing the Nernst equilibrium potential across the inner calyx membrane (Lim et al., 2011; Contini et al., 2012, 2017, Contini et al., 2020; Spaiardi et al., 2017, Spaiardi et al., 2020a). In addition, a resistive coupling between type-I hair cells and the calyx has been recently proposed (Contini et al., 2020). The non-quantal transmission would allow for very rapid (sub-ms) afferent signal transmission (Eatock, 2018; Curthoys et al., 2019; Contini et al., 2020) and prolonged depolarization that lasts for the duration of the stimulus without requiring excessive transmitter accumulation inside the restricted volume of the synaptic cleft. The exocytosis of a small RRP of vesicles, combined with non-quantal release, could add a rapid phasic pulse of depolarization in the calyx that quickly adapts to emphasize transient rather than sustained stimuli, which has been observed in calyceal afferents innervating type-I hair cells (Eatock and Songer, 2011).

The different synaptic properties of mature vestibular hair cells could have evolved to specialize them for transmitting different aspects of the vestibular sensory signal. The large RRP and SRP in type-II hair cells allow them to sustain the transmission of tonic signals at individual bouton synapses, important for maintaining head orientation relative to gravity and for encoding slowly varying signals such as low-frequency head movements as during walking or running (dominant frequency of head motion in the order of a few Hz; Grossman et al., 1988). The rapid release of a small RRP in type-I hair cells that coexists with a non-quantal transmission mechanism, instead of a large SRP, could specialize this large calyceal synapse for rapid transmission of high-frequency phasic signals (Eatock, 2018), which are likely to be required for signaling jerk (the onset of acceleration: Curthoys et al., 2019) and for driving the rapid vestibulo-ocular reflex.

## Data Availability Statement

The raw data supporting the conclusions of this article will be made available by the authors, without undue reservation.

## Ethics Statement

The animal study was reviewed and approved by University of Sheffield Ethical Review Committee.

## Author Contributions

PS and SJ performed the experiments, analyzed the data, and wrote the manuscript. SJ coordinated the study. All authors came up with the idea for the study and/or designed the experiments. SM and WM helped write the manuscript. All authors contributed to the article and approved the submitted version.

## Conflict of Interest

The authors declare that the research was conducted in the absence of any commercial or financial relationships that could be construed as a potential conflict of interest.

## Publisher’s Note

All claims expressed in this article are solely those of the authors and do not necessarily represent those of their affiliated organizations, or those of the publisher, the editors and the reviewers. Any product that may be evaluated in this article, or claim that may be made by its manufacturer, is not guaranteed or endorsed by the publisher.
